# Remotely monitored transcranial direct current stimulation in pediatric cerebral palsy: open label trial protocol

**DOI:** 10.1186/s12887-022-03612-8

**Published:** 2022-09-29

**Authors:** Emma A. Simpson, Catarina Saiote, Ellen Sutter, Daniel H. Lench, Chrysanthy Ikonomidou, Melissa A. Villegas, Bernadette T. Gillick

**Affiliations:** 1 Waisman Center, University of Wisconsin–Madison, 1500 Highland Avenue Room 491, Madison, Wisconsin 53706 USA; 2 Department of Rehabilitation Medicine, University of Minnesota, Minneapolis, Minnesota USA; 3 Department of Neurology, Medical University of South Carolina, Charleston, South Carolina USA; 4 Department of Neurology, University of Wisconsin-Madison, Madison, Wisconsin USA; 5 University of Wisconsin-Madison Pediatrics, Madison, Wisconsin USA

**Keywords:** Cerebral palsy, Transcranial direct current stimulation (tDCS), Remotely supervised tDCS, Non-invasive brain stimulation, Children, Motor function

## Abstract

**Background:**

Pediatric applications of non-invasive brain stimulation using transcranial direct current stimulation (tDCS) have demonstrated its safety with few adverse events reported. Remotely monitored tDCS, as an adjuvant intervention to rehabilitation, may improve quality of life for children with cerebral palsy (CP) through motor function improvements, reduced treatment costs, and increased access to tDCS therapies. Our group previously evaluated the feasibility of a remotely monitored mock tDCS setup in which families and children successfully demonstrated the ability to follow tDCS instructional guidance.

**Methods and design:**

Here, we designed a protocol to investigate the feasibility, safety, and tolerability of at-home active transcranial direct current stimulation in children with CP with synchronous supervision from laboratory investigators. Ten participants will be recruited to participate in the study for 5 consecutive days with the following sessions: tDCS setup practice on day 1, sham tDCS on day 2, and active tDCS on days 3-5. Sham stimulation will consist of an initial 30-second ramp up to 1.5 mA stimulation followed by a 30-second ramp down. Active stimulation will be delivered at 1.0 - 1.5 mA for 20 minutes and adjusted based on child tolerance. Feasibility will be evaluated via photographs of montage setup and the quality of stimulation delivery. Safety and tolerability will be assessed through an adverse events survey, the Box and Blocks Test (BBT) motor assessment, and a setup ease/comfort survey.

**Discussion:**

We expect synchronous supervision of at-home teleneuromodulation to be tolerable and safe with increasing stimulation quality over repeated sessions when following a tDCS setup previously determined to be feasible. The findings will provide opportunity for larger clinical trials exploring efficacy and illuminate the potential of remotely monitored tDCS in combination with rehabilitation interventions as a means of pediatric neurorehabilitation. This will demonstrate the value of greater accessibility of non-invasive brain stimulation interventions and ultimately offer the potential to improve care and quality of life for children and families with CP.

**Trial Registration:**

October 8, 2021(https://clinicaltrials.gov/ct2/show/NCT05071586)

## Background

An estimated 3.6 per 1,000 births in the United States are affected by stroke or brain bleeds which can lead to cerebral palsy (CP), a developmental disorder associated with motor impairment [1, 2]. While most rehabilitation approaches focus on behavioral repetition to improve gait and upper extremity function, these therapies can require extensively long practice times and may provide only limited recovery. Thus, there is a need for novel strategies which can advance time-efficient and consistent rehabilitation outcomes for this population [3, 4].

Rehabilitative therapies (e.g., intensive training of the upper extremities) are theoretically based in mechanisms of motor learning and use-dependent plasticity [5]. Non-invasive brain stimulation (NIBS) techniques such as transcranial direct current stimulation (tDCS) have the potential to accelerate this plasticity by polarizing neuronal membranes and enhancing NMDA dependent excitability [6]. This long-term potentiation like (LTP-like) plasticity may facilitate the reestablishment of corticospinal projections and reorganization of connectivity within the motor network [7]. In adults, rehabilitation approaches combined with tDCS have shown promise to improve motor function recovery and quality of life after stroke [8]. In children the outlook for improvement may be even greater.

Brain development during childhood is characterized by heightened neuroplastic potential [9] and presents a unique window within which motor rehabilitation can be maximized. Integrating rehabilitation therapies with the application of tDCS may enhance rehabilitation during this exceptionally neuroplastic period of childhood. tDCS has demonstrated promise as an intervention to increase the rate and extent of recovery across pediatric motor disorders including gait and upper extremity function [10–12]. In addition, tDCS provides a neuromodulation approach that is safe when applied within the proper parameter space, inexpensive, and portable. Laboratory-based studies have shown that in children with CP, tDCS is safe, feasible, and holds promise to improve motor function when combined with other pediatric rehabilitation interventions [13–15].

Although several studies have demonstrated improved effects of rehabilitation interventions in combination with tDCS [16], traveling to the laboratory or clinic can impose a burden on families, especially when multiple sessions are required. This demonstrates the need for remotely supervised at-home interventions. In adults, telerehabilitation, including integration of tDCS in the at-home environment, has made it possible to improve access, lower the cost, and improve compliance with interventions that require repetitive practice [17, 18]. However, little is known about the use of remote tDCS telerehabilitation in the pediatric population.

Prior to the COVID-19 pandemic, there were no studies of remotely performing tDCS for *pediatric* populations. Recently, we demonstrated the feasibility of performing, together with caregivers, remote *mock* tDCS in children in the home setting without compromising the efficiency, quality, and comfort of administration. We found that children and their caregivers were able to follow remote tDCS instructions with proper montage placement in a way that was tolerable for participants [19]. To advance the reach of teleneuromodulation in pediatric rehabilitation it is crucial to extend access to this affordable intervention with using already-established feasible mock setup procedures. This leads us to inquire further: 1) is active tDCS feasible in the home setting for pediatrics, and 2) are children able to tolerate teleneuromodulation using tDCS in the home setting? Answering these questions and gathering feedback about the experiences of children and caregivers during the procedure will allow expansion into large-scale randomized, sham-controlled clinical trials to assess delivery efficacy and reproducibility of combined teleneuromodulation and rehabilitation intervention for motor-skill rehabilitation [20]. If large-scale randomized, sham-controlled clinical trials are effective, teleneuromodulation interventions could provide access to personalized rehabilitation and could aid in overcoming mobility, financial, and other access challenges during the COVID-19 pandemic and beyond.

## Methods

This consecutive five-day study protocol will assess the feasibility, safety, and tolerability of remotely monitored active tDCS for children with CP due to perinatal stroke or brain bleed. Table 1 details the primary aims and hypotheses to be tested.

**Table 1:** Study aims and hypotheses

Aim	Hypothesis
1. Determine the feasibility of remotely supervised teleneuromodulation with active tDCS	1. Caregivers and children will correctly and reliably setup a stimulation montage with children following remote instruction over one practice session and one sham session
	2. Participants will be able to initiate and successfully complete active tDCS over repeated sessions, with increasing quality of stimulation (impedance)
2. Determine the safety and tolerability of 20 minutes of at-home active tDCS with remote monitoring	1. Children will tolerate the stimulation with no serious adverse events
	2. The repeated and guided sessions will result in increased comfort and confidence reported by the caregivers and children in performing tDCS at home

### Enrollment and recruitment

Ten children between the ages of 8 years and 17 years will be enrolled in this study after eligibility screening. Our Pediatric Neuromodulation Laboratory Database will be used to recruit participants who have previously taken part in our studies and expressed interest in future studies. Our previous tDCS studies have received a Food and Drug Administration (FDA) investigational device exemption (IDE). The University of Wisconsin-Madison Institutional Review Board (IRB) has approved this study. The IRB approval is not pending FDA approval; however, any outstanding FDA requirements/issues need to be satisfied before initiating the study. Inclusion and exclusion criteria will be reviewed by the medical director. Documentation of previous surgeries or concurrent rehabilitation therapies with associated characteristics influencing motor function will be collected if applicable to the participant.

### Inclusion and exclusion criteria

#### Inclusion criteria

Children with the following criteria will be included: 1) a radiologically confirmed perinatal brain bleed or stroke based on medical record review; 2) receptive language function to follow two-step commands by parent report; 3) greater than or equal to 10 degrees of active motion at the metacarpophalangeal joint by parent report.

#### Exclusion criteria

Children with any of the following will be excluded: 1) lack of access to the internet and a working computer (or equivalent device); 2) implanted devices contraindicated for use with tDCS; 3) neoplasm; 4) metabolic disorder; 5) epilepsy; 6) seizure within two years preceding the study; 7) acquired traumatic brain injury; 8) pregnancy; 9) indwelling metal or incompatible medical devices; 10) evidence of skin disease or skin abnormalities; 11) botulinum toxin or phenol block within six months preceding the study; 12) disorder of cellular migration and proliferation; 13) centrally acting agent.

**Fig. 1:**
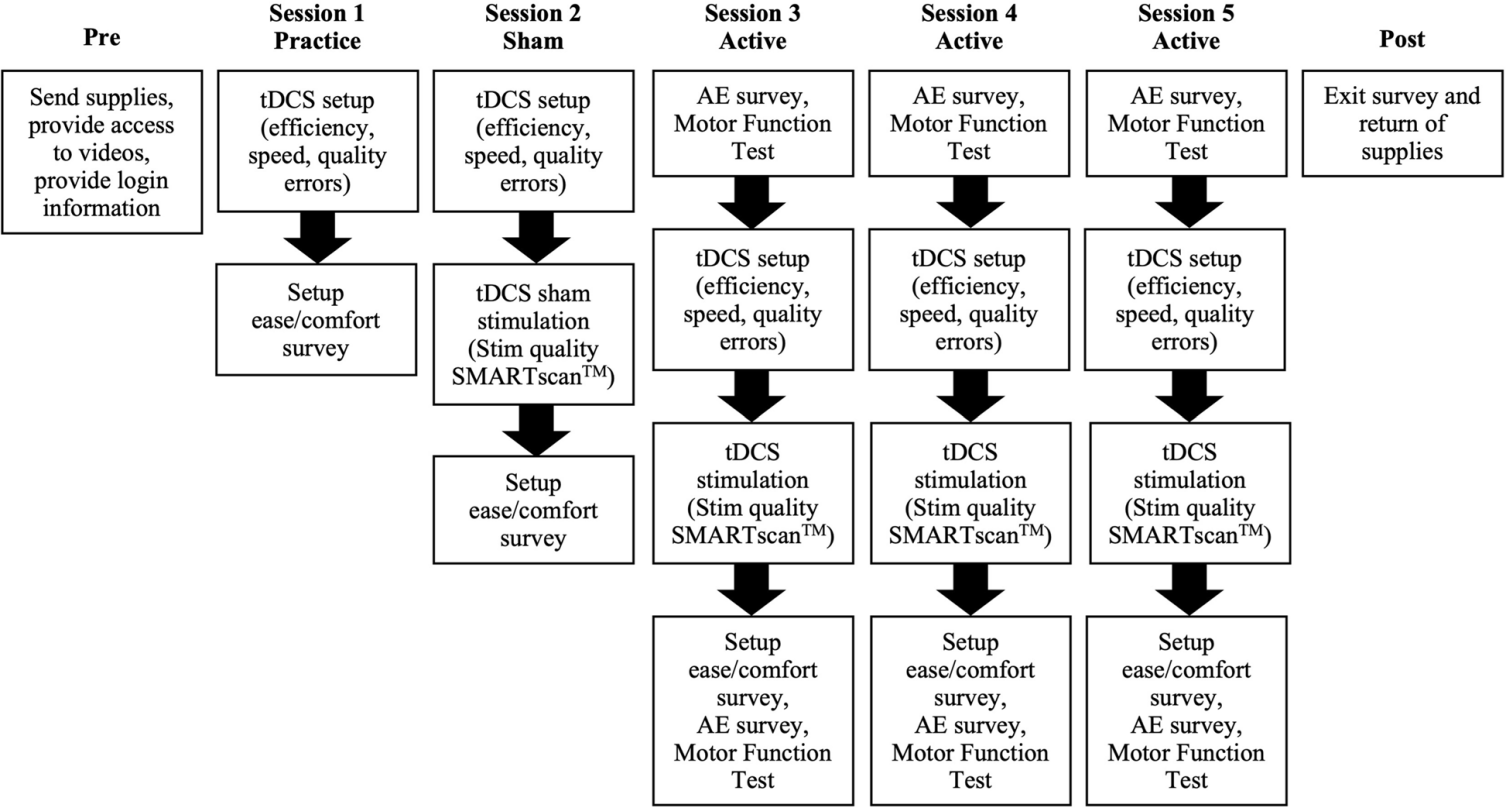
Study design. The protocol includes one setup practice session without stimulation, one sham stimulation session, and three active stimulation sessions. *AE* Adverse Events

This is an exploratory open-label unblinded serial-session study that sequentially includes one setup practice session without stimulation, one sham stimulation session, and three active stimulation sessions (Fig. 1).

### Assessments and Data Acquisition

#### Caregiver/Child Preparation

Participants will be provided with a hyperlink to the synchronous videoconferencing platform and procedure survey via email. Children and assisting parent/legal guardian – henceforth referred together as the ‘participant team’ – will receive synchronous remote supervision by the investigative team throughout the entire sessions. The participant team will receive a package of required study supplies through the mail. In preparation for study participation, a series of instructional videos will be shared that demonstrate how to initiate and complete the study procedures including 1) measuring head circumference; 2) tDCS headgear setup; 3) Box and Blocks motor function test; and 4) tDCS mini-CT device tutorial. These videos will also be embedded within the study procedure surveys for the participant team to reference. Since circadian rhythms and one’s chronotype may modulate brain function and cognitive functions [21], we will ask participants to perform tDCS stimulation on a schedule that is consistent across all 5 days.

#### tDCS device and montage

The Soterix 1x1 tDCS mini-CT will be used to deliver low-intensity tDCS in the home setting. The procedure will target the primary motor cortex (M1) as the cortical area of interest for interventions aimed at improving motor skills in our participant population. The tDCS montage will be configured with bilateral M1 electrodes with the anode over the lesioned or more-affected hemisphere (confirmed by medical record review of their neuroimaging) and the cathode on the contralateral hemisphere. This montage, which increases excitability on the anodal site and decreases excitability on the cathodal site, was selected to target hemispheric imbalance of the motor cortex that may result in maladaptive neuronal plasticity [22, 23]. Relative to studies using extracranial stimulation, one study demonstrated a reduced variability in excitability changes using bilateral tDCS montages [22]. The Soterix SNAPpad measures at 5 cm x 5 cm x 0.56 cm (depth) at saturation with an inserted 42 mm medical conductive-rubber electrode and 4 dissipation rivets.

The Soterix 1x1 tDCS mini-CT device is specifically designed to facilitate remote and regulated use of tDCS in clinical and research settings. It is equipped with separate user modes for administration and stimulation. This allows exclusive access to the authorized research staff to define stimulation parameters and constraints ahead of stimulation sessions. Participants are not able to modify the stimulation parameters and can only begin the stimulation upon receiving the corresponding activation code.

Testing and preparation of the Soterix mini-CT device to ensure it is working properly will be completed by the investigative team by following a predefined checklist, including pre-setting activation codes. Two profiles of stimulation settings will be created. The first profile will be set to a stimulation intensity of 1.5 mA (30s ramp up-down) for a duration of 20 minutes. As the tolerability of the stimulation can vary on an individual basis, we will create a second profile set to a stimulation intensity of 1.0 mA for 20 minutes in duration. This profile will be used if the higher intensity is not well tolerated by the child. Two equivalent profiles for sham stimulation will be created. For each participant, 4 unique codes will be created per profile, corresponding to the 4 active sessions. The investigators will provide the appropriate code to the participant teams at the designated time during the synchronous supervising videoconference. The battery life is expected to last up to 600 minutes of stimulation (when fully charged).

Once the montage is securely and reliably in place, and under synchronous supervision from the investigative team, the designated supervising parent/legal guardian will press the tDCS device “on” button. Next, they will access the device “stimulation mode” where the impedance of the electrode contact is identified. Participants will then iteratively adjust the electrode’s contact with the scalp until the display of contact quality reads “good” or “intermediate.” Thereafter they will enter the unique code provided by the lab team.

At any time during stimulation, the participant teams can terminate the stimulation by pressing “0” on the keypad. Once tDCS is complete, the designated supervising parent/legal guardian will be instructed to disconnect the electrodes from the child and turn off the device. The next code will not be available until the next synchronous remote session. There will be no other means by which the participant team will be allowed to turn on the device and administer active stimulation.

#### Procedure

##### tDCS setup

On all five days of the study, the participant team will be instructed to 1) place the tDCS headgear on the child’s head with the arrow aligned with the nose, make a mark on the skin with a pen/pencil under the arrow and upload an image; 2) mark locations on the skin below the red and black markers with a pen/pencil; 3) use alcohol pads to clean the skin at the marked locations and identify any notable skin status such as redness, cuts, pimples; 4) snap on the two tDCS saline saturated sponge pads to the red and black markers and upload an image; 5) connect the cables of the device using the banana plugs on the back of the device. The caregiver and laboratory team will record the time needed to complete these steps to measure the efficiency of setup.

##### Setup practice (Day 1)

Participants will be asked to leave the tDCS setup in place for 20 minutes without stimulation or turning the stimulator on.

##### Sham stimulation (Day 2)

When the tDCS electrodes are confirmed as positioned correctly, sham tDCS will be delivered consisting of a 30-second ramp-up to 1.5 mA intensity followed immediately by a 30-second ramp-down to 0 mA. This will allow the participant to briefly experience the stimulation and allow the laboratory team to adjust the intensity for active stimulation sessions if the child is unable to tolerate 1.5 mA. Participants will be asked to leave the headgear on for the 20-minute duration of the sham tDCS session.

##### Active stimulation (Day 3-5)

When the tDCS electrodes are confirmed as positioned correctly, active tDCS will be delivered at a current intensity of 1.0 – 1.5 mA (based on child tolerance) and will proceed for the 20-minute duration in each of the three active stimulation sessions. The current will ramp up from 0 mA to the desired intensity followed by a ramp down at the end of the stimulation session. Caregivers (in person) and the investigative team (remotely) will observe the child during this time to see if the electrode sponges move or if the child attempts to adjust the headgear.

### Setup ease and comfort survey

After the 20-minute duration, on all 5 days, the caregiver will be instructed to mark on the skin the new location of the tDCS headgear arrow and measure the distance it may have moved during the 20-minute duration. The survey will evaluate the overall experience of the participant team by asking the caregiver 1) how difficult the tDCS setup instructions were to understand, and 2) how instructions could be improved in the future. The survey will ask the child to describe 1) any sensations experienced while wearing the cap, and 2) how wearing the cap could be made more comfortable.

#### tDCS Safety

In this protocol, tDCS will be used up to 1.5 mA in intensity for 20 minutes. In previous studies, tDCS within these parameters was shown to be safe, feasible, tolerable, and effective in hand motor function improvement when combined with other rehabilitation methods, including constraint-induced movement therapy (CIMT) [13, 24]. Previously reported minor adverse events related to tDCS administration include transient skin irritation, discomfort, and redness at the electrode sites (Table 2). To our knowledge, though, no serious adverse events have been reported with tDCS administration [33]. We will include risk mitigation procedures established in past pediatric studies as a precautionary measure to minimize the potential for minor adverse events to occur (Table 3).

**Table 2:** Adverse events reported in studies with tdcs administration at various intensities and ages

Study	Stimulation Intensity and Duration	Age	Adverse Events Reported
Furubayashi et al., (2008) [25]	Long duration (1.0 mA, 10 minutes) or short duration (100 ms, 1.0. 3.0 or 5.0 mA)	29-50 years	None
Brunoni et al., (2011) [26]	Not Reported	Mean age of 33.5 years	Itching (39.3% of active group), tingling (10.4% of active group)
Madhavan and Shah, (2012) [27]	Not Reported	Adults	Mild tingling, moderate fatigue, itching sensation under electrodes
Gillick et al., (2015) [24]	0.7 mA, 10 minutes	7-18 years	None
Gillick et al., (2018) [13]	0.7 mA, 20 minutes	Mean age of 12.7 years	Headache, itching
Rich et al., (2018) [11]	1.5 mA, 20 minutes	7-21 years	Unusual feelings on the skin of the head
Moliadze et al., (2015) [28]	1.0 mA or 0.5 mA, 10 minutes	Mean age of 13.9 years	Itching sensation
Grecco et al., (2014) [10]	1.0 mA, 20 minutes	6-10 years	Redness and tingling of the skin
Duarte et al., (2014) [29]	1.0 mA, 20 minutes	5-12 years	Redness at cathode site, tingling sensation
Moura et al., (2017) [30]	1.0 mA, 20 minutes	6-12 years	None observed
Saleem et al., (2019) [12]	0.7 mA-2mA, 9 minutes-20 minutes	4-21 years	Tingling, discomfort, itching, skin redness
Inguaggiato et al., (2019) [31]	1.5 mA, 20 minutes	10-28 years	Transient mild headache, tingling, itchiness
Van De Winckel et al., (2018) [32]	1.5 mA, 20 minutes	Mean age of 61 years	Mild tingling sensation at electrode sites
Lench et al., (2020) [19]	Mock stimulation	11-16 years	Tightness, headache

**Table 3:** tDCS Anticipated Risks and Risk Mitigation

Anticipated Risks	Risk Mitigation
Burn- Electrolysis	Ensure proper electrode contact with skin
Stimulation in subjects with reduced sensation	Assess sensation, avoid placing electrodes over areas of decreased sensation
Stimulation over broken skin, reduced resistance	Assess skin integrity, avoid placement of electrodes over recent shaving, skin defects
Stimulation over conductive implants	Screen appropriately for exclusion criteria of implants
Stimulation over a tumor which may alter metabolic activity	Screen appropriately for exclusion criteria of neoplasm
Threshold altering pharmacologic agent	Screen appropriately for exclusion criteria of centrally acting agent
Itching, Tingling, Burning Sensation in the area of the electrodes	Ensure proper contact of surface electrodes with skin. Maintain current dosage within low-range of researched dosages. Ensure that electrode sponges are properly sanitized and that saline solution is appropriately employed.
Headache	Ensure that headband securing electrodes is in proper placement, yet not to the level of impingement of scalp area. Maintain current dosage within low range of delivery.
Pain- Neck, Scalp	Ensure that electrodes are in proper contact with skin and adjust head position as needed for comfort
Skin Redness	Ensure proper electrode position and proper level of moisture to even stimulation across the electrode
Fatigue, Sleepiness	Screen for continuous effect at follow-up visit
Concentration or Mood Changes	Evaluate cognitive status through physician examination

During stimulation, the investigator will ask the child about concerns or discomfort. If discomfort is present, the investigator will ask the child to rate the intensity from 1-10 (10 as the highest intensity). Based on previous research, if the child rates the intensity a 7 or higher or wishes to discontinue to stimulation, the stimulation will be stopped [17]. If the child were to experience discomfort rated at a 7 or higher, the caregiver and laboratory staff will opt to stop the active stimulation, assess the event, discuss with the study Medical Monitor and reassess. If deemed appropriate by the Medical Monitor the laboratory investigative team would discuss the plan to move forward with the participant team and adjust stimulation intensity for future sessions for comfort, as needed.

### Adverse events

Tolerance and report of any symptoms will be formally assessed using tDCS-specific participant safety questionnaires before and after stimulation. Assessment areas common mild effects like skin tingling and itching, as well as others such as headache, anxiety and mood changes, or any other events. Participants will rate each effect as either absent, mild, moderate, or severe. The investigator will subsequently determine the relation of the symptom to the tDCS intervention (unrelated, unlikely, possible, probable, or definite). This safety assessment has been used in our previous studies for in-person pediatric CP tDCS intervention.

### Motor Function Test

#### Box and Blocks Test (BBT)

Hand motor function will be assessed as a safety measure before and after stimulation for the active stimulation sessions (days 3-5). The child will be instructed to use one hand to move as many as possible of the 150 1-inch wooden blocks from one side of the partitioned box to the other side within 1 minute. There will be 1 trial period and 4 subsequent tests of the dominant and non-dominant hands (2 tests per hand).

Several previous intervention studies for pediatric CP have integrated the BBT due to its simple design and reliability as a measure of gross manual dexterity. In children with CP, the test-retest reliability of BBT of both the more and less-affected hand was found to be high, with an intraclass correlation coefficient of 0.95 and 0.85 respectively [34].

### Data Analysis

We will report all results according to the CARE guidelines as outlined in the Equator network.

#### tDCS montage setup efficiency

The time required to complete the tDCS montage setup will measure setup efficiency. A Friedman test will determine changes in setup speed over all sessions. All tests with a p-value of <0.05 will be considered significant.

#### tDCS setup quality

The quality of performance will be assessed by rating photographs of tDCS montage setup on a scale from 0 to 2 (“0” = incomplete, “1” = completed but incorrect, “2” = completed correctly). The photographs evaluate the participants’ ability to align the arrow on the tDCS montage with the nasion of the nose, snap on two electrode sponge pads to the head-strap with scalp contact, and connect the electrodes to the mini-CT device. The stability of the montage position will be reported as the total displacement of its position (cm) from the start of the session to the end of the session. This will be performed by having the child’s caregiver mark the scalp with a skin-safe marker at a specified location denoted by an arrow on the headgear.

#### tDCS stimulation

The Soterix Medical mini-CT device will display the contact quality (impedance) and record the number of episodes an atypical resistance (poor or open circuit) occurs, plus the associated time in the condition. The total time of stimulation will also be recorded. Descriptive statistics of the amount of time spent in each impendence category (poor, moderate, good) will be reported. A Friedman test will determine changes in stimulation quality (impedance) over the three active sessions. All tests with a p-value of <0.05 will be considered significant. We will evaluate the comfort of the child using written and verbal feedback.

We will monitor the time-of-day stimulation is delivered across study sessions; chronotype may influence tDCS induced plasticity and motor learning [21]. In an exploratory analysis, we will evaluate the effect of time of day of tDCS stimulation on our outcomes.

#### Adverse Events

The type, severity, and frequency (number of episodes) of adverse events will be reported in a descriptive table for each participant. The frequency of adverse events from this study will be compared with the frequency of adverse events reported in previous tDCS studies in pediatrics populations (see Table 2).

#### Box and Blocks Test

A one-way ANOVA or non-parametric Friedman’s test will be used to evaluate changes in the number of blocks moved between partitions. The significance level will be set at *p* < 0.05.

## Discussion

The protocol and assessments of our study are designed carefully to ensure optimal quality, ease, and tolerability of tDCS stimulation. In our previous study, we identified successful procedures for connecting with participants remotely. We found that it was feasible for participants to complete tDCS protocol setup successfully with the use of REDCap surveys and instructional videos. All of the assessments have been effectively used in previous in-person or remote intervention studies.

Guidelines for at home tDCS for the adult population from Charvet et al, 2020 [17] were incorporated into our at-home tDCS protocol for the pediatric population. Specifically, with safety given the utmost importance, we are following the Study Stop Criteria guidelines which include specific criteria for each stage of study session participation: initiating teleconferencing and remote supervision, adverse event reporting and collection, electrode preparation, electrode placement, engaging stimulation, during stimulation session and session completion [17]. Furthermore, our study design incorporating practice, sham, and three active stimulation session allows for gradual training and stimulation intensity adaptation as needed to refine quality and tolerability throughout study participation. Three days of active stimulation will replicate the repeated stimulation often paired with rehabilitation therapies in clinical trials. Lastly, presence of study team members over videoconferencing platforms during all sessions allows for continued guidance, safety monitoring, and provides the opportunity for participants to ask questions.

While the protocol is designed to refine the tolerability, ease, quality, and comfort of tDCS stimulation, the proposed study does have limitations. The intended sample size of 10 limits data analysis and will not permit generalization to all patients with CP seeking interventions. Instead, this study is designed for an initial assessment of safety and tolerability before expanded testing. Also, this study requires accessibility to the internet and a computer, or a device with similar function, which may create a barrier for participation. In addition, the requirement for synchronous videoconferencing with simultaneous data collection via an online survey may lead to unanticipated difficulties for the participants. Plans for an expanded trial should consider providing a computer/tablet device to participants to facilitate participation.

This study protocol is designed to explore the feasibility, safety, and tolerability of pediatric tDCS in the home setting building upon feasible mock setup study procedures and previous in-person active tDCS studies. The findings from this study will set the foundation for future larger clinical trials evaluating the delivery efficacy of at-home combined tDCS and rehabilitation, harnessing both neuroplasticity and motor skills towards improved motor function. Integrating tDCS as an adjuvant modality to telerehabilitation has the potential to enhance therapy efficacy and to offer improved remote intervention access, potentially reducing both treatment cost and burden in cases of limited access to clinic or hospital facilities. This study will also inform future study protocols that investigate processes and safety of continuing pediatric teleneuromodulation in the home setting. Safe and effective tDCS in the home setting may accommodate for access challenges to non-invasive brain stimulation interventions, and subsequently increase the access to personalized rehabilitation for children with CP.

## Data Availability

The datasets used and/or analyzed during the current study are de-identified and will be available from the corresponding author on reasonable request.

## Abbreviations

AE: Adverse events
BBT: Box and Blocks Test
CIMT: Constraint induced movement therapy
CP: Cerebral palsy
FDA: Food and Drug Administration
IDE: Investigational Device Exemption
NIBS: Non-invasive brain stimulation
tDCS: transcranial direct current stimulation
